# A multimodal biological margin risk index predicts recurrence after neoadjuvant immunochemotherapy in head and neck squamous cell carcinoma

**DOI:** 10.3389/fimmu.2026.1740643

**Published:** 2026-02-06

**Authors:** Ning Xu, Defeng Chen, Junui Yuan, Tao Huang, Xu Zhang, Qigen Fang, Wenlu Li

**Affiliations:** 1 Special Clinic, Henan Provincial Stomatological Hospital, The First Affiliated Hospital of Zhengzhou University, Zhengzhou, China; 2 Department of Head Neck and Thyroid, The Affiliated Cancer Hospital of Zhengzhou University & Henan Cancer Hospital, Zhengzhou, China; 3 Department of Radiology, The Affiliated Cancer Hospital of Zhengzhou University & Henan Cancer Hospital, Zhengzhou, China; 4 Department of Breast, The Affiliated Cancer Hospital of Zhengzhou University & Henan Cancer Hospital, Zhengzhou, China; 5 Department of Stomatology, The First Affiliated Hospital of Zhengzhou University, Zhengzhou, China

**Keywords:** head and neck squamous cell carcinoma, immunotherapy, neoadjuvant therapy, prognosis, surgical margin

## Abstract

**Background:**

Conventional classification of surgical margins is inadequate for head and neck squamous cell carcinoma (HNSCC) treated with neoadjuvant immunochemotherapy (NICT), as it fails to capture the complex biological changes in the tumor microenvironment. This study aimed to develop a novel definition of a negative margin.

**Methods:**

We conducted a retrospective analysis of treatment-naïve, HPV-negative HNSCC patients who completed NICT followed by surgery. Surgical margins underwent multi-modal assessment, including histopathology (tertiary lymphoid structures), tumor burden (Pan-CK, Ki-67), molecular profiling (driver mutations, PD-L1 RNA), and immune contexture (CD8+/FoxP3+ ratio, Granzyme B). A Margin Risk Index (MRIx) was developed by weighting these domains based on their prognostic impact for locoregional control (LRC) and distant metastasis-free survival (DMFS). The MRIx was externally validated in an independent cohort.

**Results:**

The study included a training cohort of 144 patients and an independent validation cohort of 100 patients. The MRIx integrated four domains into a continuous score, stratifying patients into low, intermediate, and high-risk categories. The MRIx significantly outperformed traditional margin assessment, with superior discrimination for both LRC (C-index=0.72) and DMFS (C-index=0.75). External validation confirmed its prognostic power, demonstrating significant risk stratification (log-rank p<0.001 for both LRC and DMFS) and an independent hazard ratio for high-risk patients (HR = 2.95 for LRC; HR = 3.22 for DMFS, both p<0.001).

**Conclusion:**

The proposed MRIx provides a biologically-grounded tool that redefines margin status following NICT. It accurately identifies patients at high risk of recurrence who may benefit from treatment intensification and those with low-risk margins suitable for de-escalation, enabling personalized adjuvant therapy.

## Introduction

Head and neck squamous cell carcinoma (HNSCC) is a prevalent and aggressive malignancy, with surgical resection remaining a cornerstone of curative treatment (1). The status of surgical margins is a critical determinant of postoperative outcomes, influencing both local recurrence rates and overall survival. Traditional margin assessment defines clear margins (R0) as the absence of tumor cells at the resection edge, with close or positive margins (R1/R2) necessitating adjuvant therapies or re-resection (2). However, these definitions were established in the context of upfront surgery and may not fully account for the biological and pathological changes induced by neoadjuvant therapies, particularly immunochemotherapy.

Recent advances in neoadjuvant immunochemotherapy (NICT) have revolutionized the management of locally advanced HNSCC, offering tumor downstaging, enhanced immune activation, and improved resectability (3, 4). Despite these benefits, the histological and molecular alterations in tumor margins following NICT remain poorly understood. Conventional margin assessment methods, such as frozen section analysis and histopathological evaluation, may fail to capture the complex tumor-immune interactions and residual microscopic disease in this setting (5). Moreover, the current binary classification of margin status (positive/negative) does not reflect the heterogeneous responses to NICT, where residual tumor cells may exhibit altered biological behavior or immune-mediated dormancy (6).

This paper proposes a novel definition of negative margin status tailored to HNSCC patients treated with NICT, integrating histopathological, molecular, and immunological parameters. By redefining margin assessment in this context, we aim to provide a more accurate prognostic tool and guide personalized adjuvant strategies.

## Patients and methods

### Ethical compliance and study design

**Figure 1:**
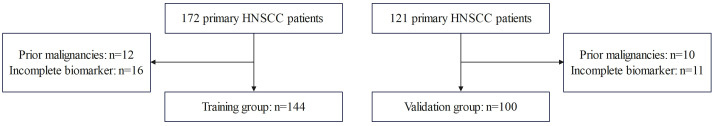
Flowchart of patient selection for the training and validation cohorts.

This study was conducted in strict accordance with ethical guidelines after receiving approval from the Institutional Review Board of Henan Cancer Hospital. Written informed consent was obtained from all participants following the principles outlined in the Declaration of Helsinki. The research protocol was designed to comply with REMARK guidelines for biomarker studies to ensure methodological rigor and transparency (7). We performed a retrospective analysis from 172 treatment-naïve HNSCC patients (excluding HPV-related HNSCC) treated between 2019-2022, all of whom completed NICT followed by curative surgery. Patients with prior malignancies (n=12) or incomplete biomarker data (n=16) were excluded, resulting in a final cohort of 144 patients (**Figure 1**). This design enabled comprehensive evaluation of treatment responses while maintaining robust clinical and pathological correlation.

### Tissue processing and multi-modal assessment

Immediately following resection based on residual tumor, surgical specimens underwent standardized processing within 30 minutes to preserve tissue integrity. The tumor bed and 5mm radial margins were meticulously oriented using a 6-color inking system (Tissue Marking Dyes, Cancer Diagnostics, Durham, NC, USA) and photographed under 4K resolution for spatial documentation (Canon EOS R5, Canon Inc., Tokyo, Japan). Tissue aliquots were prepared for multi-omic analysis: OCT-embedded fragments were snap-frozen in liquid nitrogen vapor phase (CryoStar NX70 Cryostat, Thermo Fisher Scientific, Waltham, MA, USA) for molecular studies, while matched samples were fixed in 10% neutral-buffered formalin (Sigma-Aldrich, St. Louis, MO, USA) (24h, 4°C) with vacuum-assisted processing (Tissue-Tek VIP 6, Sakura Finetek, Torrance, CA, USA) for optimal histomorphology. Serial sectioning was performed using cryostat (Leica CM1950, Leica Biosystems, Wetzlar, Germany) (5μm for IHC) and microtome (Leica RM2255, Leica Biosystems) (4μm for FFPE) under RNase-free conditions. This protocol ensured compatibility across histopathological, molecular, and spatial biology platforms while minimizing pre-analytical variables. (**Supplementary Table 1**).

### Histopathological and immunohistochemical evaluation

Conventional histopathological assessment served as the foundation for margin evaluation, with dual-blinded analysis to grade treatment effects. Special attention was given to fibrosis maturity, tertiary lymphoid structure (TLS) density (CD23+/CD21+ networks), and tumor-immune interface geometry. Automated IHC on the Ventana Benchmark Ultra platform (Roche Diagnostics, Basel, Switzerland) employed validated antibodies including pan-CK (AE1/AE3, 1:100) for tumor identification, CD8 (C8/144B, RTU) for cytotoxic T-cells, and PD-L1 (22C3 pharmDx, Dako) for immune checkpoint assessment. Digital pathology analysis using HALO AI (Indica Labs, Albuquerque, NM, USA) v3.4 enabled quantitative evaluation of ≥100 high-power fields per case, providing objective measures of immune cell infiltration patterns and tumor regression features.

### Molecular profiling and spatial biology

For DNA analysis, a multiplex PCR approach followed by Sanger sequencing validation was performed. This method focused on core HNSCC driver mutations in TP53 (exons 5-8) and NOTCH1 (exon 34). The protocol began with DNA extraction from 5μm FFPE curls using the QIAamp DNA FFPE kit (Qiagen, Hilden, Germany). Multiplex PCR amplification was performed using QIAGEN Multiplex PCR Plus reagents with the following primer sequences:

TP53 Exon 5: Forward 5’-TTC CTT ACT GCC TCC TGC TC-3’, Reverse 5’-CAG CCC TGT CGT CTC TCC AG-3’

TP53 Exon 6: Forward 5’-GAG GCC ACT GAC AAC CAC CA-3’, Reverse 5’-CAC TGA CAA CCA CCC CTT AA-3’

TP53 Exon 7: Forward 5’-CTG GCA TCT TGG GCC TGT GT-3’, Reverse 5’-AGG GGT CAG AGG CAA GCA GG-3’

TP53 Exon 8: Forward 5’-CCT ATC CTG AGT AGT GGT AA-3’, Reverse 5’-CCT GCT TGC TTA CCC TCG CT-3’

NOTCH1 Exon 34: Forward 5’-CTG CCT GTC CTC ACC TCA TC-3’, Reverse 5’-GAG GTG AGG AGC AGG AAG AG-3’

The PCR products were purified and sequenced on an ABI 3500xl system (Applied Biosystems, Foster City, CA, USA). Sequences were analyzed with Mutation Surveyor software (SoftGenetics, State College, PA, USA). To corroborate the correctness of sequencing, the CAL-27 cell line DNA (known TP53 p.R175H mutation) and the SCC-25 cell line DNA (known NOTCH1 p.R2319 mutation) were included as positive controls in each run. A no-template control and DNA from normal lymphocytes were included as negative controls.

For RNA expression analysis, a focused RT-qPCR panel in 96-well format was implemented. The panel included essential immune markers (CD8A, PDCD1, CD274, FOXP3), EMT markers (VIM, CDH1), and reference genes (GAPDH, ACTB) using the following pre-designed TaqMan assays (Thermo Fisher Scientific, Waltham, MA, USA): CD8A (Hs00233520_m1), PDCD1 (Hs01550088_m1), CD274 (Hs00204257_m1), FOXP3 (Hs01085834_m1), VIM (Hs00958111_m1), CDH1 (Hs01023894_m1), GAPDH (Hs02786624_g1), and ACTB (Hs01060665_g1). Starting with RNA extraction from FFPE samples using the RNeasy FFPE kit (Qiagen), followed by cDNA synthesis with the High-Capacity cDNA Reverse Transcription Kit (Applied Biosystems). qPCR was performed on a QuantStudio system (Applied Biosystems). To control for assay performance, cDNA from activated T-cells (for immune markers) was used as a positive control. A no-reverse transcription control and a no-template control were included in each experiment to rule out genomic DNA contamination and non-specific amplification, respectively.

For spatial analysis of the tumor microenvironment, a sequential immunofluorescence method using the Opal 7-color system (Akoya Biosciences, Marlborough, MA, USA) was proposed. This strategy employed two staining cycles: the first detects pan-CK (tumor), CD8 (cytotoxic T-cells), and DAPI, while the second examines PD-L1, FoxP3, and DAPI. The protocol involved standard antigen retrieval with pH9 buffer, overnight primary antibody incubation at 4°C, Opal fluorophore conjugation, and microwave stripping between cycles, with final imaging on a Vectra system (Akoya Biosciences) at 20x magnification.

For image analysis, open-source tools like QuPath for cell segmentation and the free version of HALO for basic spatial analysis was utilized. Key metrics include CD8+ cell density within 100μm of tumor nests and percentage of PD-L1+ tumor cells, which provide sufficient microenvironment characterization for most clinical correlation studies.

### Study variables

Tumor and nodal staging were determined in accordance with the 8^th^ edition of the AJCC staging system (8). Pathological differentiation was classified into well-differentiated, moderately differentiated, or poorly differentiated categories (9). Surgical margin distance was divided into close (≤5mm) and clear (>5mm) margin (10). Major pathologic response (mPR) was defined as ≤10% residual viable tumor cells in the primary tumor bed and all sampled lymph nodes, assessed through standardized histopathologic examination of hematoxylin and eosin–stained sections by two blinded pathologists. Pathologic complete response (pCR) was defined as the complete absence of viable tumor cells (0%) in both the primary tumor site and all resected lymph nodes, with evidence of treatment-related fibrosis or inflammation (11). The combined positive score (CPS) was utilized to appraise the proportion of PD-L1-positive tumor and infiltrating immune cells in relation to the total viable tumor cells. Radiological responses were evaluated in adherence with the Response Evaluation Criteria in Solid Tumors version 1.1 (12).

The study’s primary endpoints were rigorously defined as: (1) locoregional control (LRC), measured from surgery date to first histologically confirmed recurrence in the primary tumor bed or regional lymph nodes (censored at last follow-up for recurrence-free patients), and (2) distant metastasis-free survival (DMFS), calculated from surgery date to radiologically confirmed distant metastasis or the last follow-up.

### Treatment

The NICT regimen consisted of docetaxel at a dosage of 75 mg/m², cisplatin at 75 mg/m², and pembrolizumab or alternative PD-1 inhibitors at 200 mg per three-week cycle for two to three cycles. Treatment plans and resection margins were established based on residual tumor. Adjuvant therapy was initiated within six weeks post-surgery, focusing on the tumor bed with a 1-2 cm margin. After curative therapy, standardized surveillance included clinical examination and contrast-enhanced CT/MRI of the head/neck/chest at 3-month intervals for the first 2 years, every 6 months until year 5, and annually thereafter. Suspicious findings triggered additional diagnostic workup including PET/CT or biopsy. All recurrence events were independently adjudicated by a oncology endpoint review committee comprising a radiologist, pathologist, and treating surgeon, with discrepancies resolved by consensus.

### Statistical analysis

The statistical analysis plan was designed to comprehensively evaluate the association between multimodal margin assessment and clinical outcomes. Continuous variables were presented as medians with ranges and compared using the Mann-Whitney U test or Kruskal-Wallis test, as appropriate. Categorical variables were expressed as frequencies with percentages and analyzed using Pearson’s χ² test or Fisher’s exact test for small sample sizes. Time-to-event outcomes including LRC and DMFS were calculated using the Kaplan-Meier method, with differences between groups assessed by the log-rank test. Multivariable Cox proportional hazards regression models were constructed to adjust for potential confounding factors including age, sex, clinical stage, and pathological response, with results reported as hazard ratios (HR) and 95% confidence intervals (CI). The discriminative ability of the Margin Risk Index (MRIx) was evaluated using using Harrell’s Concordance Index (C-index). The relative quality of nested models was evaluated using the Akaike Information Criterion (AIC), with a lower value indicating a preferable balance of model fit and complexity. The improvement in model fit afforded by the addition of the MRIx was formally tested using the Likelihood Ratio Test (LRT). All statistical tests were two-sided, and a p-value <0.05 was considered statistically significant. Analyses were performed using R software (version 4.2.2) with the survival, and survminer packages. Multiple testing correction was applied using the Benjamini-Hochberg method for exploratory biomarker analyses to control the false discovery rate at 5%.

### External validation

The generalizability of the MRIx was assessed through an external validation study using an independent cohort of HNSCC patients who underwent NICT followed by surgical resection at The First Affiliated Hospital of Zhengzhou University. This validation cohort was retrospectively enrolled and included patients treated between January 2021 and December 2022. The cohort was processed and analyzed using the identical standardized protocols for tissue handling, multi-omics assessment, and scoring criteria as defined in this study.

External validation of the MRIx was performed through a multi-faceted analytical approach. First, discriminatory power was assessed by evaluating the ability of the pre-defined MRIx risk categories (Low, Intermediate, High) to stratify patients using Kaplan-Meier survival analysis for LRC and DMFS, with group comparisons performed by the log-rank test. Second, the overall predictive accuracy of the continuous MRIx score was quantified using Harrell’s Concordance Index (C-index) for both LRC and DMFS. Third, the independent prognostic value of the MRIx was confirmed by constructing multivariable Cox proportional hazards regression models within the validation cohort, adjusting for key clinicopathological variables. Finally, calibration assessment was conducted by plotting calibration curves comparing the predicted versus observed incidence of recurrence at a clinically relevant time point, with the Hosmer-Lemeshow goodness-of-fit test used for statistical evaluation. Successful validation was defined by significant separation of Kaplan-Meier curves, a C-index meaningfully above 0.5, a significant independent hazard ratio for the MRIx in multivariable analysis, and a calibration curve aligning closely with the line of perfect agreement.

## Results

This training cohort of 144 patients with HNSCC was predominantly male (69.4%) and over 55 years of age (54.9%). The majority of tumors exhibited positive PD-L1 expression (CPS≥1, 66.7%) and were of moderate or poor differentiation (70.1%). Most patients presented with clinical stage III disease (61.8%). Following NICT, the radiologic assessment showed a high response rate, with 86.1% of patients achieving either a complete (29.2%) or partial response (56.9%). Pathologic evaluation correlated with this, revealing that 72.2% of patients experienced a mPR, including 31.9% with a pCR. Adverse pathologic features such as perineural invasion (6.9%), lymphovascular invasion (6.3%), and extranodal extension (2.8%) were relatively uncommon. A clear surgical margin was achieved in the vast majority of cases (93.7%) (**Table 1**).

**Table 1:** Baseline data of the patients in training group (n=144) and validation cohort (n=100).

Variable	Training group (n=144)	Validation cohort (n=100)	p
Age			0.432
≤55	65 (45.1%)	40 (40.0%)	
>55	79 (54.9%)	60 (60.0%)	
Sex			0.470
Male	100 (69.4%)	65 (65.0%)	
Female	44 (30.6%)	35 (35.0%)	
Primary site			0.027
Oral/oropharynx	97 (67.4%)	80 (80.0%)	
Larynx/hypopharynx	47 (32.6%)	20 (20.0%)	
CPS			0.371
<1	48 (33.3%)	28 (28.0%)	
≥1	96 (66.7%)	72 (72.0%)	
Differentiation			0.781
Well	43 (29.9%)	27 (27.0%)	
Moderate	56 (38.9%)	44 (44.0%)	
Poor	45 (31.2%)	29 (29.0%)	
Clinical stage			0.070
III	89 (61.8%)	73 (73.0%)	
IV	55 (38.2%)	27 (27.0%)	
Radiologic response			0.403
CR	42 (29.2%)	36 (36.0%)	
PR	82 (56.9%)	54 (54.0%)	
SD	20 (13.9%)	10 (10.0%)	
Pathologic response			0.937
pCR	46 (31.9%)	34 (34.0%)	
mPR but not pCR	58 (40.3%)	38 (38.0%)	
No-mPR	40 (27.8%)	28 (28.0%)	
PNI			0.538
No	134 (93.1%)	95 (95.0%)	
Yes	10 (6.9%)	5 (5.0%)	
LVI			0.723
No	135 (93.7%)	95 (95.0%)	
Yes	9 (6.3%)	5 (5.0%)	
Extranodal extension			1.000
No	140 (97.2%)	97 (97.0%)	
Yes	4 (2.8%)	3 (3.0%)	
Margin			0.916
Close	9 (6.3%)	6 (6.0%)	
Clear	135 (93.7%)	94 (94.0%)	

CR, complete response; PR, partial response; SD, stable disease; pCR, pathologic complete response; mPR, major pathologic response; PNI, perineural invasion; LVI, lymphovascular invasion

Of the 144 patients in the training cohort, with a median follow-up of 42 months (range: 24-72 months) from the time of surgery to the last follow-up point in July 2025, a total of 26 (18.1%) locoregional recurrence events and 31 (21.5%) distant metastasis events were recorded.

Several clinicopathological variables significantly associated with oncologic outcomes. Tumor differentiation and pathologic response were the most powerful predictors for both LRC and DMFS. Patients with poorly differentiated tumors had significantly worse LRC and DMFS compared to those with well or moderate differentiation (p<0.001 for both). Similarly, patients without a mPR faced a significantly higher risk of both locoregional recurrence and distant metastasis compared to those achieving a major or complete response (p<0.001 for both). Furthermore, surgical margin status was a significant prognostic factor, as patients with close margins had inferior LRC (p=0.005) and DMFS (p=0.049) compared to those with clear margins. In contrast, factors including patient age, sex, primary tumor site, perineural invasion, lymphovascular invasion, and extranodal extension did not demonstrate a statistically significant association with either LRC or DMFS in this cohort (**Supplementary Table 2**).

Histopathologically, a robust TLS response (≥3 TLS/mm²) was present in 35.4% of patients, while 20.8% exhibited a paucity of TLS formation (<1 TLS/mm²). 31.9% of margins had no detectable cytokeratin-positive (Pan-CK+) cells, whereas 27.8% harbored substantial residual disease (≥5 Pan-CK+ foci/mm²). Highly proliferative residual cells (Ki-67 >20%) existed in 11.8% of margins. 13.9% of samples harbored multiple driver mutations alongside significant PD-L1 RNA overexpression (>2-fold). Conversely, 47.2% of margins were cleared of detectable driver mutations. There was a favorable cytotoxic-to-suppressive ratio (CD8+/FoxP3+ ≥2) and high cytotoxic activity (Granzyme B+ >200 cells/mm²) in 37.5% and 34.7% of patients, respectively. However, a low CD8+/FoxP3+ ratio (<1) and minimal cytotoxic activity (Granzyme B+ <50 cells/mm²) was evident in approximately one-fifth of cases (20.1% and 19.4%, respectively) (**Table 2**).

**Table 2:** Multi-modal assessment of the margin.

Domain	Number (%)
Histopathologic	
≥3 TLS/mm²	51 (35.4%)
1-2 TLS/mm²	63 (43.8%)
<1 TLS/mm²	30 (20.8%)
Tumor burden	
No detectable Pan-CK+ cells	46 (31.9%)
1–4 Pan-CK+ foci/mm²	58 (40.3%)
≥5 Pan-CK+ foci/mm²	40 (27.8%)
Ki-67 <5%	75 (52.1%)
Ki-67 5–20%	52 (36.1%)
Ki-67 >20%	17 (11.8%)
Molecular	
Absence of driver mutations	68 (47.2%)
Single mutation	56 (38.9%)
≥2 mutations	20 (13.9%)
PD-L1 RNA expression <1-fold	59 (41.0%)
PD-L1 RNA 1–2-fold elevation	62 (43.1%)
PD-L1 >2-fold overexpression	23 (16.0%)
Immune*	
CD8+/FoxP3+ ratio ≥2	54 (37.5%)
CD8+/FoxP3+ ratio 1–1.9	61 (42.4%)
CD8+/FoxP3+ ratio <1	29 (20.1%)
Granzyme B+ >200 cells/mm²	50 (34.7%)
Granzyme B+ 50–200 cells/mm²	66 (45.8%)
Granzyme B+ <50 cells/mm²	28 (19.4%)

* the ratio referred to CD8+/FoxP3+ ratio.

**Figure 2:**
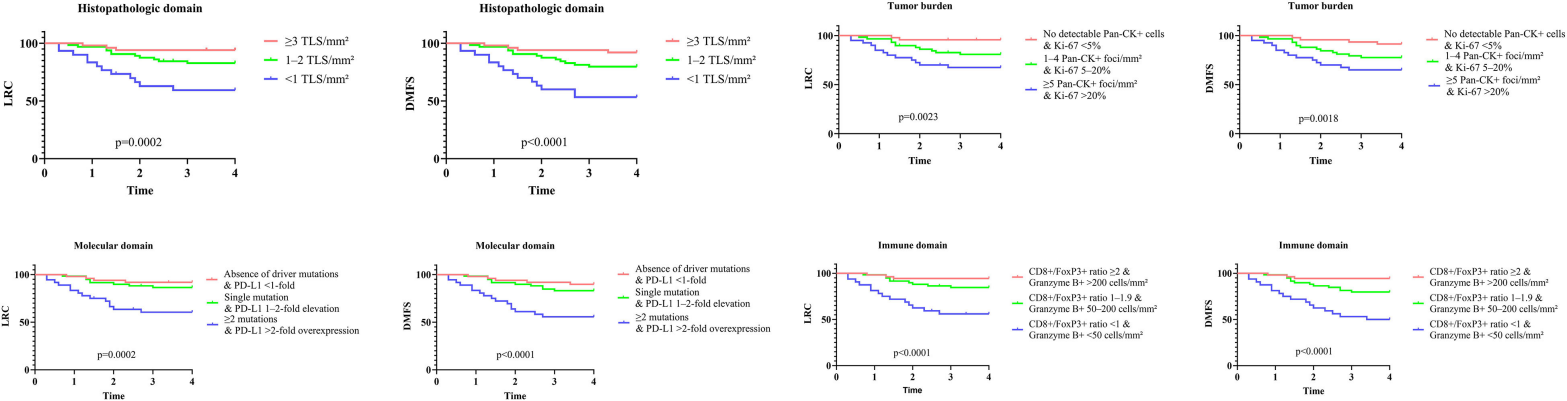
Impact of histopathologic, tumor burden, molecular, and immune domains on locoregional control (LRC) and distant metastasis free survival (DMFS).

The four prognostic domains were each stratified into three risk levels (scores 0-2). In histopathologic domain, low-risk margins (score=0) were defined by a high density of TLS ≥3/mm². Intermediate risk (score=1) was defined as 1–2 TLS/mm², while high-risk margins (score=2) exhibited a complete absence of TLS. In tumor burden domain, low-risk margins (score=0) showed no detectable Pan-CK^+^ cells and a Ki-67 index of <5%. Intermediate risk (score=1) was characterized by 1–4 Pan-CK^+^ foci/mm² and a Ki-67 index of 5–20%. High-risk margins (score=2) contained ≥5 Pan-CK^+^ foci/mm² and a Ki-67 index >20%. In molecular domain: a low-risk status (score=0) required the absence of driver mutations and PD-L1 RNA expression below the level of normal mucosa (<1-fold). Intermediate risk (score=1) was defined by a single driver mutation with PD-L1 expression 1–2 times that of normal mucosa. High-risk status (score=2) involved ≥2 driver mutations combined with PD-L1 overexpression >2-fold above normal. In immune contexture domain, a low-risk profile (score=0) required a CD8^+^/FoxP3^+^ ratio ≥2 and >200 Granzyme B^+^ cells/mm². An intermediate status (score=1) showed a ratio of 1–1.9 and 50–200 Granzyme B^+^ cells/mm². A high-risk profile (score=2), marked by T-cell exhaustion, was defined by a ratio <1 and <50 Granzyme B^+^ cells/mm². Significant associations were observed between all four domains and both LRC and DMFS (**Figure 2**; all p<0.05).

To create a clinically actionable MRIx, we systematically evaluated four critical biological domains that collectively predict recurrence risk. Each domain was weighted according to its relative prognostic impact, derived from multivariate Cox regression analysis of our 144-patient cohort (**Supplementary Table 3**). The histopathologic domain, encompassing TLS density, was assigned the greatest weight (30%) as it served as the most robust foundational predictor, with a high-risk TLS absence conferring a striking 5.92-fold and 6.75-fold increased risk for locoregional recurrence and distant metastasis, respectively. The tumor burden and molecular MRD domains were each prioritized at 25%, balancing their critical yet distinct roles. The high-risk tumor burden category was the strongest predictor of locoregional failure (HR = 7.11), while the high-risk molecular MRD profile—defined by multiple driver mutations and PD-L1 overexpression—was the single most powerful predictor of distant metastasis (HR = 9.40). Finally, the immune contexture domain received a slightly lower weight (20%), as its high-risk exhausted phenotype was a consistent significant predictor (HR = 3.80 for LRC; HR = 4.25 for DMFS), but its intermediate risk category did not reach statistical significance. These individual domain scores are then integrated using the formula: MRIx Score = (Histopathology × 0.3) + (Tumor Burden × 0.25) + (Molecular MRD × 0.25) + (Immune Context × 0.2).

The resulting composite MRIx score stratifies patients into three clinically actionable categories: low-risk (0–0.8), intermediate-risk (0.9–1.4), and high-risk (1.5–2.0). The presence of a surgical margin (≤2mm) was considered an automatic indicator of high risk, overriding the numerical score (**Table 3**).

**Table 3:** Multivariable analysis of predictors for locoregional control (LRC) and distant metastasis free survival (DMFS) based on the Margin Risk Index (MRIx).

Variable	LRC		DMFS	
	HR [95%CI]	p	HR [95%CI]	p
Differentiation				
Well	ref		ref	
Moderate	1.56 [0.74-5.36]	0.216	1.62 [0.68-5.78]	0.187
Poor	2.19 [1.36-5.65]	0.009	2.00 [1.11-5.78]	0.021
Pathologic response^				
pCR	ref		ref	
mPR but not pCR	1.45 [0.55-4.46]	0.439	1.92 [0.69-5.25]	0.319
No-mPR	2.06 [1.34-5.43]	0.012	1.78 [1.19-5.45]	0.033
MRIx				
Low-risk (0–0.8)	ref		ref	
Intermediate-risk (0.9–1.4)	1.89 [1.17-4.65]	0.007	2.18 [1.32-6.46]	0.005
High-risk (1.5–2.0)	3.15 [2.04-9.87]	<0.001	3.54 [2.16-10.97]	<0.001

^ pCR: pathologic complete response; mPR: major pathologic response;

The quantitative superiority of the MRIx over traditional margin assessment was demonstrated in **Table 4**. The model incorporating only clinical factors (differentiation and pathologic response) showed modest discrimination for LRC (C-index=0.60) and DMFS (C-index=0.65). The addition of traditional binary margin status to create a second model failed to provide a statistically significant improvement in predictive performance for either endpoint (**Supplementary Table 4**), as indicated by non-significant Likelihood Ratio Tests (LRC: LRT χ²=3.7, p=0.054; DMFS: LRT χ²=3.6, p=0.058) and minimal increases in the C-index. In stark contrast, integrating the MRIx into the clinical model resulted in a dramatic and highly significant enhancement for predicting both LRC and DMFS. The MRIx-based model demonstrated significantly superior discrimination (LRC C-index=0.72; DMFS C-index=0.75) and a substantially better fit, evidenced by significant Likelihood Ratio Tests against both the baseline clinical model (LRC: χ²=30.4, p<0.001; DMFS: χ²=29.6, p<0.001) and the traditional margin model (LRC: χ²=26.7, p<0.001; DMFS: χ²=26.0, p<0.001), alongside a marked reduction in AIC values.

**Table 4:** Comparison of Cox model performance for predicting locoregional control (LRC) and distant metastasis-free survival (DMFS).

Model	LRC			
	C-index	AIC	LRT vs Model 1	LRT vs previous model
Clinical	0.60 [0.54-0.66]	422.7		
Clinical+traditional margin	0.61 [0.55-0.67]	421.0	3.7/0.054	3.7/0.054
Clinical+MRIx	0.72 [0.67-0.77]	392.3	30.4/<0.001	26.7/<0.001
	DMFS			
	C-index	AIC	LRT vs Model 1	LRT vs previous model
Clinical	0.65 [0.59-0.71]	440.1		
Clinical+traditional margin	0.66 [0.60-0.72]	438.5	3.6/0.058	3.6/0.058
Clinical+MRIx	0.75 [0.70-0.80]	410.5	29.6/<0.001	26.0/<0.001

C-index, concordance index; AIC, Akaike Information Criterion; LRT, Likelihood Ratio Test; Clinical refers to Differentiation and pathologic response.

The LRT compares the goodness-of-fit between two nested models. A significant p-value (p < 0.05) indicates that the more complex model provides a statistically significant improvement.

An independent validation cohort of 100 HNSCC patients was recruited. The cohort’s baseline characteristics were comparable to the development cohort, with a similar distribution of age, sex, clinical stage, and pathologic response (**Table 1**).

**Figure 3:**
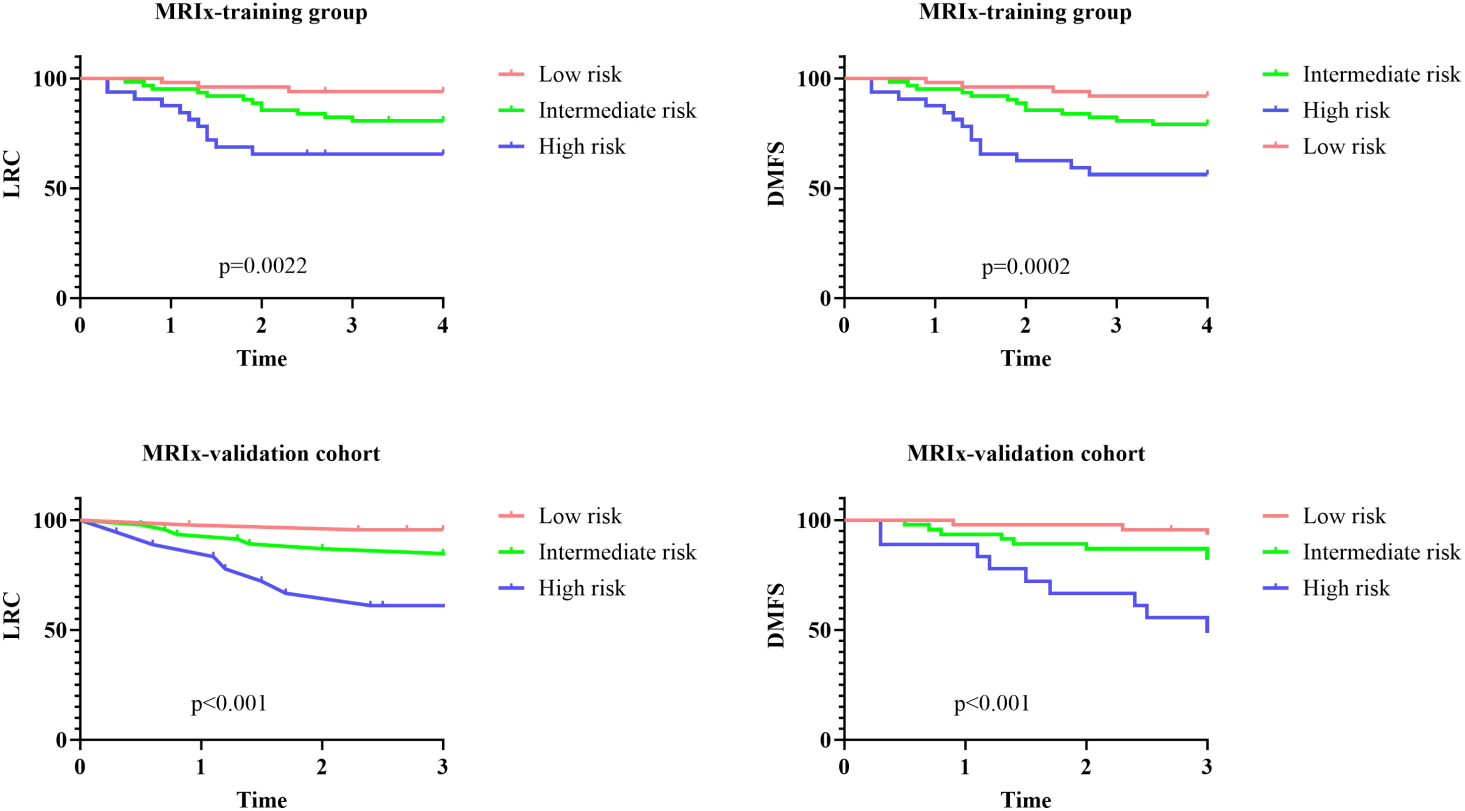
Impact of margin risk index (MRIx) on locoregional control (LRC) and distant metastasis free survival (DMFS) in training and validation groups.

The pre-defined MRIx successfully stratified patients in the external cohort by demonstrating significant separation between the three risk categories for both LRC (log-rank p<0.001) and DMFS (log-rank p<0.001) (**Figure 3**). The MRIx exhibited strong and preserved discriminatory power, with a Harrell’s Concordance Index (C-index) of 0.67 (95% CI: 0.60-0.74) for LRC and 0.70 (95% CI: 0.63-0.77) for DMFS.

**Figure 4:**
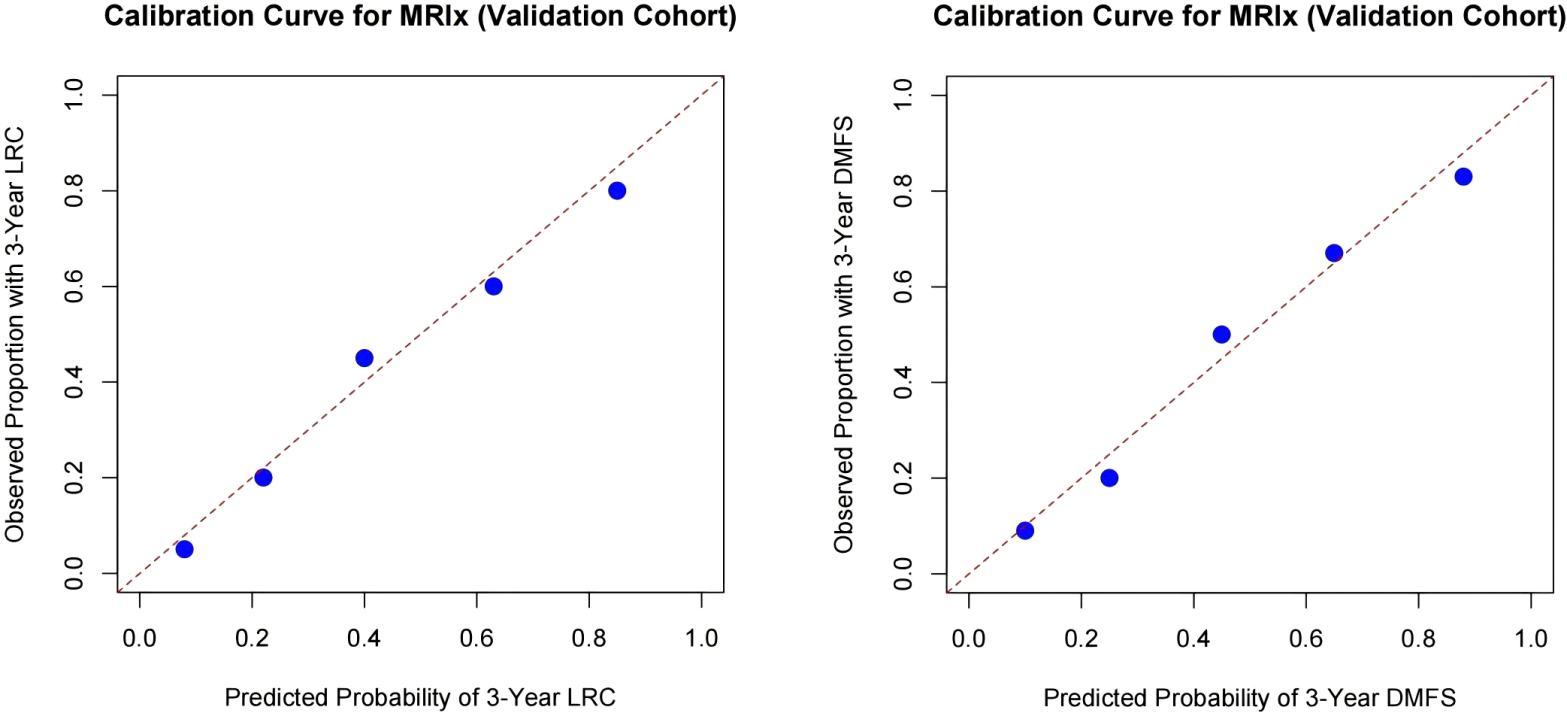
Accuracy of margin risk index (MRIx) in predicting locoregional control (LRC) and distant metastasis free survival (DMFS).

Crucially, the MRIx retained its independent prognostic value after adjusting for established clinical factors. The MRIx high-risk category remained a powerful predictor of both inferior LRC (HR = 2.95, 95% CI: 1.65-6.25, p<0.001) and DMFS (HR = 3.22, 95% CI: 1.85-8.60, p<0.001), after adjusting for tumor differentiation, pathologic response, and traditional margin status (**Supplementary Table 5**). The calibration curve for 3-year LRC and DMFS showed acceptable agreement between predicted and observed outcomes, with a nonsignificant Hosmer-Lemeshow test result (both p>0.05), indicating adequate model calibration (**Figure 4**).

## Discussion

Our study develops and validates a novel MRIx that redefines surgical margin assessment for HNSCC following NICT. By synthesizing histopathological, tumor burden, molecular, and immunological features into a weighted, continuous score, the MRIx significantly outperforms traditional binary margin classification. It provides superior discrimination for both LRC (C-index=0.72) and DMFS (C-index=0.75), offering a robust tool to identify patients at high risk of recurrence who may benefit from treatment intensification and those with low-risk margins suitable for de-escalation.

Surgical margin status remains one of the most critical prognostic determinants in HNSCC. Positive margins are consistently associated with a significantly elevated risk of locoregional recurrence, distant metastasis, and reduced overall survival, invariably necessitating aggressive adjuvant therapy (13). The assessment of margins is complicated by technical factors such as post-resection tissue shrinkage and the type of surgical instrumentation used. Conventionally, margins are categorized as involved (<1 mm), close (1–5 mm), or clear (>5 mm) based on the distance of tumor cells from the resection edge (14). While the adverse impact of a positive margin is unequivocal, the clinical significance of a close margin remains a subject of considerable debate. A comprehensive meta-analysis of 26 studies demonstrated that clear margins were associated with a significantly lower risk of 5-year local recurrence (RR 0.50, 95% CI 0.38–0.65) and higher overall survival (RR 1.22, 95% CI 1.11–1.35) compared to close margins (15). Involved margins, in turn, predicted a higher risk of local recurrence compared to close margins (RR 1.75, 95% CI 1.16–2.64). This is supported by a recent study of 342 patients which found that in tongue SCC, both positive (HR 13.48, 95% CI 2.03–32.91) and close margins (HR 3.87, 95% CI 1.31–11.34) were independent predictors of local recurrence, suggesting a threshold of concern may be <4 mm for this subsite (16). However, the prognostic weight of a close margin appears context-dependent, influenced by other clinicopathological factors. A multicenter analysis found that for pN+ patients with close margins, adjuvant therapy did not confer a significant improvement in 3-year disease-specific survival (DSS) (51.9% surgery alone vs. 53.2% with adjuvant therapy) (17). Instead, factors such as T-classification, lymphovascular space invasion (LVSI), and extranodal extension were more dominant predictors of outcome. Further complicating the definition, a study of 244 patients found that while an involved margin (<1 mm) predicted worse DSS (p=0.04), a margin threshold of <3 mm was a more powerful independent predictor on multivariate analysis, significantly associated with local control (p=0.01), DSS (p=0.007), and overall survival (p=0.005) (18). This suggests that the current 5 mm standard for a “clear” margin may be ideal but that a 3 mm threshold provides more robust prognostic stratification, highlighting the inadequacy of a simple binary classification. The inability of contemporary studies to consistently establish a significant association between close margins and prognosis underscores a critical flaw in our current paradigm of margin evaluation. The core issue lies in the imprecision of anatomic localization; the process of relocating a specific margin intraoperatively or *post-hoc* based on surgeon labels is profoundly variable, with demonstrated mean errors exceeding 1 cm (19). This variability in sampling and interpretation likely contaminates cohort data, obscuring the true biological risk of close margins and fueling ongoing clinical controversy. This growing recognition has led to the consensus that traditional histopathological assessment alone is an inadequate tool for guiding adjuvant therapy decisions.

Several efforts have been made to better evaluate the margin status. A prominent strategy involves the use of near-infrared fluorescence-guided surgery (NIR-FGS), where molecularly targeted agents like panitumumab-IRDye800CW (anti-EGFR) are administered intravenously to illuminate tumor tissue intraoperatively. This technique has demonstrated significant potential in improving the identification of positive margins and detecting satellite lesions that are missed by conventional white-light visualization and palpation (20, 21). Complementing this, research into novel molecular targets beyond EGFR aims to improve specificity; for example, TACNA profiling has identified GLUT-1 and P-cadherin as highly overexpressed in HNSCC, suggesting they could yield higher tumor-to-background ratios and reduce non-specific background fluorescence in future imaging applications (20). In parallel, advanced optical imaging technologies are being developed for ex vivo specimen analysis. Hyperspectral imaging (HSI), which captures a wide spectrum of light to characterize tissue composition, has been integrated with deep learning algorithms, to objectively classify cancerous and normal margins on excised tissue. This approach leverages both spectral and spatial information, achieving high diagnostic accuracy (AUC up to 0.94 for thyroid cancer and 0.86 for HNSCC) and providing a quantitative, digital pathology-like assessment that could supplement or even reduce reliance on frozen section analysis (22). Despite these technological advances, several limitations impede widespread clinical adoption. The penetration depth of NIR light remains constrained to approximately 5 mm, limiting its ability to assess deep margins or nodes buried in tissue. Furthermore, fluorescence signal intensity can be influenced by variable agent concentration, tissue autofluorescence, and prior treatments such as radiation, which may induce fibrosis and alter biodistribution. There is also a critical need for standardization of imaging protocols, quantification methods, and device interoperability across surgical centers to enable reproducible implementation and validation in multi-institutional trials.

Some authors advocate for the adjunctive use of immunohistochemistry for markers such as p53/TP53 and PERP to refine risk stratification in patients with histologically negative mucosal margins. The overexpression or aberrant accumulation of p53, a hallmark of TP53 gene mutation, has demonstrated significant prognostic value. The specificity of p53 immunohistochemistry in predicting locoregional recurrence was reported as 0.844 (95% CI: 0.78–0.89; p < 0.001), with a positive likelihood ratio of 3.032 (95% CI: 2.17–4.22; p < 0.001) and a negative likelihood ratio of 0.487 (95% CI: 0.35–0.67; p < 0.001). This indicates that p53 positivity in a histologically clear margin is a strong predictor of recurrence. The relative risk of locoregional recurrence in p53-positive margins was 3.13, supported by a significant odds ratio of 5.249 (95% CI: 3.176–8.676; p < 0.001). A subgroup meta-analysis further revealed that this predictive capability was particularly pronounced in laryngeal squamous cell carcinoma (p < 0.001), underscoring the site-specific utility of this biomarker (23). Similarly, the loss of PERP, a p53-apoptosis related protein, has been associated with adverse outcomes. In a study of 44 patients (24), the 2-year cumulative incidence of local relapse was 44.4% in the PERP-negative group compared to 16.4% in the PERP-positive group (p = 0.01). A trend toward worse progression-free survival (p = 0.09) and overall survival (p = 0.06) was also observed with loss of PERP expression, suggesting its role as a potential suppressor of tumor recurrence and a valuable marker for identifying high-risk margins that appear benign by conventional histology. These molecular techniques highlight a critical evolution in margin assessment, moving beyond morphological analysis to incorporate functional cellular states. The integration of such biomarkers could potentially identify a subset of “molecularly positive” margins that warrant closer surveillance or adjuvant therapy, ultimately aiming to improve long-term oncologic control.

Due to confirmed survival benefit of NICT in HNSCC (25), concept of response-adapted surgery attracts extensive attention (26). For instance, a Neoadjuvant Immuno-Radiotherapy Trial involving 21 HNSCC patients reported promising outcomes: after neoadjuvant therapy, most underwent resection based on post-treatment tumor size, with only two requiring adjuvant therapy. At a median follow-up of 472 days, all patients were alive, and 20 (95%) showed no evidence of disease. Two patients experienced lymph node recurrence—one in the contralateral neck and another in the ipsilateral level 5—both successfully treated with salvage neck dissection. A third patient (p16-negative oropharyngeal cancer) recurred in the tongue base but achieved SD with immunotherapy at data cutoff (27). Cao et al. (28) evaluated 111 HNSCC patients treated with NICT followed by reduced surgery, reporting an objective response rate of 77.5% and a pCR rate of 40.5%. Notably, 72.1% avoided flap reconstruction, and 70.8% of initially planned mandibulectomies were spared. With a median follow-up of 27 months, the 3-year EFS and OS rates were 80.9% and 91.3%, respectively. In a matched-pair analysis (29), patients receiving NICT followed by limited surgery were compared to those undergoing upfront surgery. The NICT group exhibited a mPR rate of 58.8% and an objective response rate of 66.7%. These patients also experienced shorter operative times, reduced hospitalization, less blood loss, and fewer flap reconstructions (p < 0.001). Additionally, they reported significantly better QoL in sensory function, speech, social eating, social contact, and overall well-being (p < 0.05). Critically, there were no significant differences in OS (p = 0.825) or disease-free survival (p = 0.473) between groups. Collectively, these results validate the feasibility of de-escalation surgery after NICT. This paradigm shift underscores two critical considerations: the rarity of positive margins owing to consistent tumor regression, and the fact that resections are now frequently performed within the post-treatment tumor bed. Consequently, the traditional anatomical definition of a margin is obsolete, necessitating a new biological definition for the immunotherapy era. Our study directly addresses this critical question. The MRIx provides a biologically-grounded framework to redefine margin status in this new context. It moves beyond the anatomical distance of residual tumor cells to assess the biological potential of the tissue remaining *in situ*. A margin with minimal residual disease that is molecularly quiescent and immunologically active (low MRIx score) may be considered truly negative, even if located within the original tumor bed. Conversely, a margin that is histologically “clear” by traditional standards but exhibits a high-risk molecular and exhausted immune profile (high MRIx score) identifies a patient who may benefit from intensified adjuvant therapy despite a technically successful resection. This is because in responding patients, while there is a profound decrease of active regulatory T-cell population and intratumoral dysfunctional CD8+ T cells displayed decreased expression of activity and dysfunction-related genes (30), our data show that a residual exhausted immune microenvironment and persistent oncogenic drivers at the margin can still portend a high risk of recurrence. Thus, the MRIx offers a nuanced tool to guide post-surgical management, enabling surgeons to confidently de-escalate adjuvant therapy in low-risk patients while identifying those with high-risk biological features who require further intervention, ultimately personalizing care in the era of NICT.

Despite the robust multi-modal design and external validation, this study has several limitations. First, while the cohort size is substantial for a detailed biomarker study, it originates from a single institution, and the external validation, though critical, was conducted at a single center within the same geographic region. Future multi-institutional prospective studies are warranted to confirm the generalizability of the MRIx across diverse populations and practice settings. Second, the comprehensive molecular and spatial biology analyses, while highly informative, are resource-intensive and may present challenges for immediate widespread clinical adoption. Efforts to streamline the assay into a more cost-effective and automated diagnostic platform are necessary for broader implementation. Third, as with any retrospective study, there is potential for selection bias, despite our efforts to mitigate this through prospective data collection and predefined exclusion criteria. The decision-making for adjuvant therapy was based on standard clinicopathological factors and not the MRIx, which could influence outcomes; a prospective trial where adjuvant therapy is guided by the MRIx is the logical next step to definitively establish its clinical utility. Finally, the exclusion of HPV-related HNSCC means the findings are primarily applicable to HPV-negative HNSCC, and the performance of the MRIx in HPV-driven cancers remains an important area for future investigation.

In conclusion, the era of NICT necessitates a paradigm shift in how we define oncologic surgical success. The traditional anatomical binary of “positive” or “negative” margins is insufficient to capture the complex biological landscape of the post-NICT tumor microenvironment. Our proposed MRIx integrates histopathological, tumor burden, molecular, and immunological features into a novel, biologically-grounded, and continuous prognostic tool. Validated both internally and externally, the MRIx significantly outperforms conventional margin assessment, providing superior discrimination for recurrence risk. This framework moves beyond mere anatomic clearance to assess the biological potential of the tissue left behind, offering a powerful strategy to personalize post-surgical management. By accurately identifying patients with low-risk biological margins who can safely avoid aggressive adjuvant therapy and those with high-risk features who may benefit from treatment intensification, the MRIx paves the way for a more precise and effective approach to achieving oncologic control in HNSCC.

## Data Availability

The original contributions presented in the study are included in the article/**Supplementary Material**. Further inquiries can be directed to the corresponding author.
