# Supplementary material for: A multimodal biological margin risk index predicts recurrence after neoadjuvant immunochemotherapy in head and neck squamous cell carcinoma
Source: Front Immunol. 2026 Feb 6;17:1740643. doi: 10.3389/fimmu.2026.1740643 (PMC12920489; doi:10.3389/fimmu.2026.1740643)
Supplement: Supplementary file 1 [file Table1.doc]

****Supplementary Table 1. Antibodies, Assay Conditions, and Controls Used in Multi-modal Assessment****

| ****Assay Type**** | ****Target**** | ****Clone / Assay ID**** | ****Source**** | ****Dilution / Format**** | ****Positive Control**** | ****Negative Control**** |
| --- | --- | --- | --- | --- | --- | --- |
| ****IHC**** | Pan-CK | AE1/AE3 | Thermo Fisher Scientific | 1:100 | Known positive squamous cell carcinoma tissue | Omission of primary antibody; mouse IgG1, κ isotype control |
|  | CD8 | C8/144B | Roche Diagnostics | Ready-to-use (RTU) | Tonsil tissue | Omission of primary antibody |
|  | PD-L1 | 22C3 pharmDx | Dako (Agilent) | RTU kit | PD-L1 high-expressing tumor tissue | Kit-provided negative control tissue |
| ****DNA Sequencing**** | TP53 (exons 5–8) | Custom primers | Laboratory designed | N/A | CAL-27 cell line DNA (p.R175H mutation) | No template control (NTC); normal lymphocyte DNA |
|  | NOTCH1 (exon 34) | Custom primers | Laboratory designed | N/A | SCC-25 cell line DNA (p.R2319* mutation) | No template control (NTC); normal lymphocyte DNA |
| ****RT-qPCR (RNA)**** | CD8A, PDCD1, CD274, FOXP3, VIM, CDH1 | Pre-designed TaqMan assays | Thermo Fisher Scientific | Assay-specific | Activated T-cell cDNA (high expression) | No-RT control; no template control (NTC) |
| ****Multiplex Immunofluorescence (mIHC)**** | Pan-CK | AE1/AE3 | Thermo Fisher Scientific | 1:100 (Opal 520) | Squamous cell carcinoma tissue | Mouse IgG1, κ isotype control |
|  | CD8 | C8/144B | Roche Diagnostics | RTU (Opal 570) | Tonsil tissue | Omission of primary antibody |
|  | PD-L1 | 22C3 | Dako (Agilent) | RTU (Opal 620) | PD-L1-positive tumor tissue | Kit-provided negative control |
|  | FoxP3 | 236A/E7 | Abcam | 1:50 (Opal 690) | Lymph node Treg region | Rat IgG2a, κ isotype control |

****Notes:****

All IHC and mIHC staining was performed on the Ventana Benchmark Ultra platform (Roche Diagnostics) following manufacturer protocols.

For mIHC, the Opal 7-color kit (Akoya Biosciences) was used with sequential staining, antigen retrieval (pH9), and microwave stripping between cycles.

DNA and RNA extraction was performed using QIAamp DNA FFPE and RNeasy FFPE kits (Qiagen), respectively.

Sanger sequencing was carried out on an ABI 3500xl system (Applied Biosystems).

RT-qPCR was performed using TaqMan assays on a QuantStudio system (Thermo Fisher Scientific).
