# Supplementary material for: A multimodal biological margin risk index predicts recurrence after neoadjuvant immunochemotherapy in head and neck squamous cell carcinoma
Source: Front Immunol. 2026 Feb 6;17:1740643. doi: 10.3389/fimmu.2026.1740643 (PMC12920489; doi:10.3389/fimmu.2026.1740643)
Supplement: Supplementary file 2 [file Table2.doc]

Supplementary Table 2. Univariate analysis of predictors for locoregional control (LRC) and distant metastasis free survival (DMFS).

| Variable | LRC | DMFS |
| --- | --- | --- |
| Age |  |  |
| ≤55 |  |  |
| >55 | 0.643 | 0.896 |
| Sex |  |  |
| Male |  |  |
| Female | 0.342 | 0.578 |
| Primary site |  |  |
| Oral/oropharynx |  |  |
| Larynx/hypopharynx | 0.265 | 0.339 |
| Differentiation |  |  |
| Well |  |  |
| Moderate |  |  |
| Poor | <0.001 | <0.001 |
| Pathologic response^ |  |  |
| pCR |  |  |
| mPR but not pCR |  |  |
| No-mPR | <0.001 | <0.001 |
| Perineural invasion |  |  |
| No |  |  |
| Yes | 0.114 | 0.676 |
| Lymphovascular invasion |  |  |
| No |  |  |
| Yes | 0.206 | 0.457 |
| Extranodal extension |  |  |
| No |  |  |
| Yes | 0.996 | 0.999 |
| Margin |  |  |
| Close |  |  |
| Clear | 0.005 | 0.049 |

^ pCR: pathologic complete response; mPR: major pathologic response;
