# Supplementary material for: A multimodal biological margin risk index predicts recurrence after neoadjuvant immunochemotherapy in head and neck squamous cell carcinoma
Source: Front Immunol. 2026 Feb 6;17:1740643. doi: 10.3389/fimmu.2026.1740643 (PMC12920489; doi:10.3389/fimmu.2026.1740643)
Supplement: Supplementary file 3 [file Table3.doc]

Supplementary Table 3. Multivariable analysis of predictors for locoregional control (LRC) and distant metastasis free survival (DMFS).

| Variable | LRC | | DMFS | |
| --- | --- | --- | --- | --- |
|  | HR [95%CI] | p | HR [95%CI] | p |
| Differentiation |  |  |  |  |
| Well | ref |  | ref |  |
| Moderate | 1.67 [0.88-5.46] | 0.154 | 1.58 [0.76-6.26] | 0.209 |
| Poor | 2.15 [1.43-6.28] | 0.006 | 1.99 [1.14-5.66] | 0.025 |
| Pathologic response^ |  |  |  |  |
| pCR | ref |  | ref |  |
| mPR but not pCR | 1.22 [0.67-4.80] | 0.427 | 1.89 [0.74-5.13] | 0.254 |
| No-mPR | 1.96 [1.25-4.38] | 0.016 | 1.66 [1.12-4.29] | 0.046 |
| Histopathologic domain |  |  |  |  |
| TLS ≥3/mm² | ref |  | ref |  |
| 1–2 TLS/mm² | 2.85 [1.21-7.74] | <0.001 | 3.10 [1.29-7.43] | 0.011 |
| <1 TLS/mm² | 5.92 [2.65-13.22] | <0.001 | 6.75 [2.98-15.37] | <0.001 |
| Tumor burden |  |  |  |  |
| No Pan-CK+ cells/ Ki-67 <5% | ref |  | ref |  |
| 1–4 Pan-CK+/ Ki-67 5–20% | 2.50 [1.08-5.78] | 0.032 | 3.22 [1.38-7.52] | 0.007 |
| ≥5 Pan-CK+ / Ki-67 >20% | 7.11 [3.25-15.57] | <0.001 | 8.90 [4.05-19.63] | <0.001 |
| Molecular domain |  |  |  |  |
| No mutation/PD-L1<1-fold | ref |  | ref |  |
| Single mutation/PD-L1 1-2-fold | 2.10 [0.88-5.01] | 0.094 | 3.05 [1.30-7.17] | 0.011 |
| ≥2 mutations/PD-L1>2-fold | 4.65 [2.15-10.10] | <0.001 | 9.40 [4.40-20.15] | <0.001 |
| Immune domain^&^ |  |  |  |  |
| ratio≥2/Granzyme B+ >200² | ref |  | ref |  |
| ratio-1.9/Granzyme B+ 50–200 | 1.95 [0.85-4.47] | 0.114 | 2.20 [0.96-5.34] | 0.061 |
| ratio <1/Granzyme B+ <50 | 3.80 [1.75-8.25] | 0.001 | 4.25 [1.95-9.26] | <0.001 |

^ pCR: pathologic complete response; mPR: major pathologic response;

& ratio refers to CD8+/FoxP3+ ratio
