# Supplementary material for: A multimodal biological margin risk index predicts recurrence after neoadjuvant immunochemotherapy in head and neck squamous cell carcinoma
Source: Front Immunol. 2026 Feb 6;17:1740643. doi: 10.3389/fimmu.2026.1740643 (PMC12920489; doi:10.3389/fimmu.2026.1740643)
Supplement: Supplementary file 4 [file Table4.doc]

Supplementary Table 4. Multivariable analysis of predictors for locoregional control (LRC) and distant metastasis free survival (DMFS) based on traditional margin assessment.

| Variable | LRC | | DMFS | |
| --- | --- | --- | --- | --- |
|  | HR [95%CI] | p | HR [95%CI] | p |
| Differentiation |  |  |  |  |
| Well | ref |  | ref |  |
| Moderate | 1.55 [0.73-3.28] | 0.250 | 1.60 [0.75-3.41] | 0.222 |
| Poor | 2.25 [1.21-4.18] | 0.010 | 2.10 [1.12-3.94] | 0.021 |
| Pathologic response^ |  |  |  |  |
| pCR | ref |  | ref |  |
| mPR but not pCR | 1.50 [0.61-3.72] | 0.377 | 1.95 [0.79-4.81] | 0.147 |
| No-mPR | 2.15 [1.12-4.11] | 0.021 | 1.85 [1.01-3.40] | 0.047 |
| Margin |  |  |  |  |
| Close | ref |  | ref |  |
| Clear | 1.40 [0.75-6.61] | 0.291 | 1.30 [0.69-4.43] | 0.414 |

^ pCR: pathologic complete response; mPR: major pathologic response;
