# Supplementary material for: A multimodal biological margin risk index predicts recurrence after neoadjuvant immunochemotherapy in head and neck squamous cell carcinoma
Source: Front Immunol. 2026 Feb 6;17:1740643. doi: 10.3389/fimmu.2026.1740643 (PMC12920489; doi:10.3389/fimmu.2026.1740643)
Supplement: Supplementary file 5 [file Table5.doc]

Supplementary Table 5. Multivariable analysis of predictors for locoregional control (LRC) and distant metastasis free survival (DMFS) in validation group

| Variable | LRC | | DMFS | |
| --- | --- | --- | --- | --- |
|  | HR [95%CI] | p | HR [95%CI] | p |
| Differentiation |  |  |  |  |
| Well | ref |  | ref |  |
| Moderate | 1.45 [0.62-6.37] | 0.392 | 1.52 [0.65-6.53] | 0.332 |
| Poor | 2.05 [1.05-6.00] | 0.035 | 1.98 [1.14-6.88] | 0.047 |
| Pathologic response^ |  |  |  |  |
| pCR | ref |  | ref |  |
| mPR but not pCR | 1.60 [0.60-5.25] | 0.345 | 1.85 [0.70-5.89] | 0.215 |
| No-mPR | 2.15 [1.11-5.99] | 0.024 | 1.92 [1.12-6.63] | 0.044 |
| Margin status |  |  |  |  |
| Clear | ref |  | ref |  |
| Close | 1.35 [0.70-5.60] | 0.371 | 1.28 [0.66-5.48] | 0.446 |
| MRIx |  |  |  |  |
| Low-risk (0–0.8) | ref |  | ref |  |
| Intermediate-risk (0.9–1.4) | 1.80 [1.02-4.55] | 0.042 | 2.05 [1.15-6.10] | 0.015 |
| High-risk (1.5–2.0) | 2.95 [1.65-6.25] | <0.001 | 3.22 [1.85-8.60] | <0.001 |

^ pCR: pathologic complete response; mPR: major pathologic response;
